# Anticancer applications of phytochemicals in gastric cancer: Effects and molecular mechanism

**DOI:** 10.3389/fphar.2022.1078090

**Published:** 2023-01-12

**Authors:** Zhaofeng Liang, Yumeng Xu, Yue Zhang, Xinyi Zhang, Jiajia Song, Hui Qian, Jianhua Jin

**Affiliations:** ^1^ Wujin Institute of Molecular Diagnostics and Precision Cancer Medicine of Jiangsu University, Wujin Hospital Affiliated with Jiangsu University, Chang Zhou, China; ^2^ Department of Laboratory Medicine, School of Medicine, Jiangsu University, Zhenjiang, China

**Keywords:** gastric cancer, phytochemicals, prevention, treatment, mechanisms

## Abstract

Gastric cancer (GC) is the fourth most common malignant cancer and is a life-threatening disease worldwide. Phytochemicals have been shown to be a rational, safe, non-toxic, and very promising approach to the prevention and treatment of cancer. It has been found that phytochemicals have protective effects against GC through inhibiting cell proliferation, inducing apoptosis and autophagy, suppressing cell invasion and migration, anti-angiogenesis, inhibit *Helicobacter pylori* infection, regulating the microenvironment. In recent years, the role of phytochemicals in the occurrence, development, drug resistance and prognosis of GC has attracted more and more attention. In order to better understand the relationship between phytochemicals and gastric cancer, we briefly summarize the roles and functions of phytochemicals in GC tumorigenesis, development and prognosis. This review will probably help guide the public to prevent the occurrence and development of GC through phytochemicals, and develop functional foods or drugs for the prevention and treatment of gastric cancer.

## 1 Introduction

GC is the fourth most common malignant cancer and the third most common cause of cancer-related death worldwide, with more than 1 million new cases and 769000 deaths annually (Sung et al., 2021). Chemotherapy, radiotherapy and surgery have been recognized as the main therapies for the treatment of gastric cancer, but they have their own disadvantages, such as side effects, toxicity and resistance of anticancer drugs (Khan et al., 2019). In addition, GC is a multicentric and multistep phenomenon which sequentially accumulates molecular and genetic abnormalities. Therefore, it is urgent and necessary to find a multi-stage, more effective and less toxic strategy for the prevention and treatment of gastric cancer (Mao et al., 2020).

Although surgery with or without chemotherapy/radiotherapy as a standard treatment can be an appropriate treatment strategy for gastric cancer, side effects and drug resistance are the two major obstacles to therapy. It has been found that phytochemical agents exhibited significant anticancer activity while causing trivial side effects (Cheshomi et al., 2022).

**FIGURE 1:**
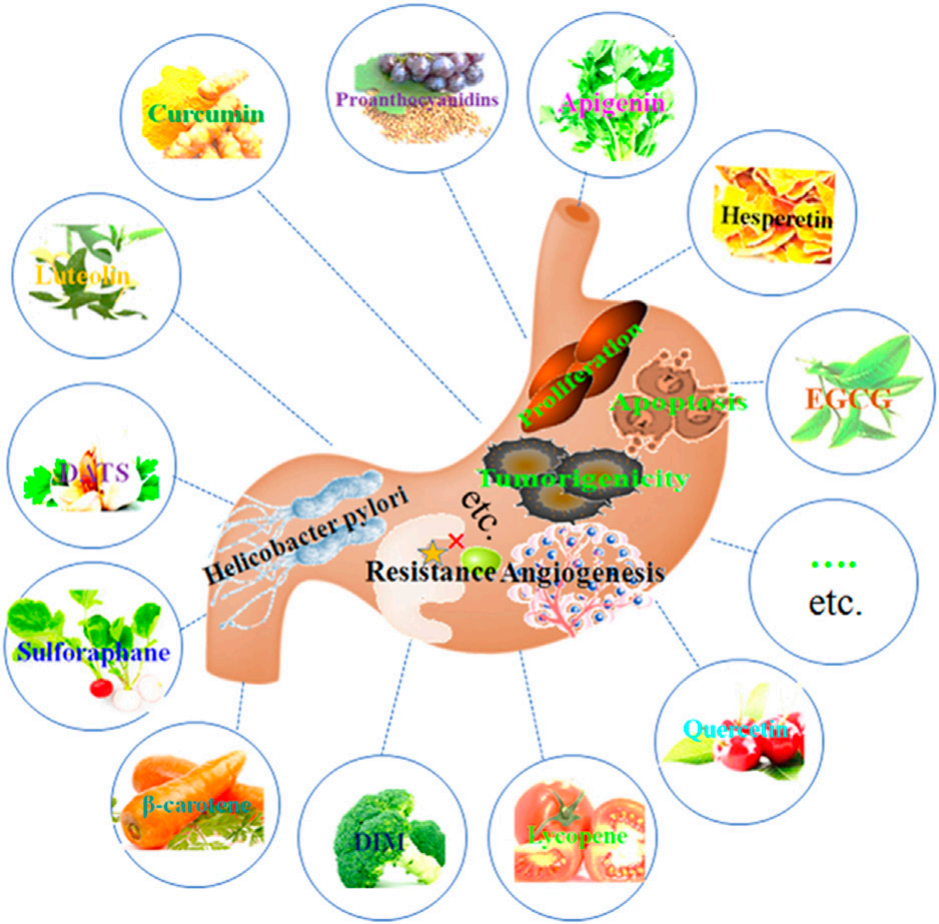
Phytochemicals have protective effects against GC through inhibiting cell proliferation, inducing cell apoptosis and autophagy, suppressing cell invasion and migration, anti-angiogenesis, inhibiting Helicobacter pylori infection, regulating microenvironment, and other possible mechanisms.

Phytochemicals have been shown to be a rational, safe, non-toxic, and very promising approach to the prevention and treatment of cancer, especially in high-risk populations (Lu et al., 2016). A rich phytochemical is found in vegetables, spices, fruits, nuts, soy, tea, edible macro-fungi and whole grains, which have a variety of health benefits (Bastos et al., 2010; Al-Ishaq et al., 2020; Mao et al., 2020). Numerous epidemiological investigations and experimental studies have demonstrated that phytochemical is essential to the prevention and management of gastric cancer (Nagata et al., 2002; Bastos et al., 2010; Mao et al., 2020). Phytochemicals have protective effects against GC through various mechanisms, including inhibiting cell proliferation, inducing cell apoptosis and autophagy, suppressing cell invasion and migration, anti-angiogenesis, inhibiting Helicobacter pylori infection, regulating the microenvironment, and other possible mechanisms (Figure 1).

The objective of this review is to summarize anti-cancer effects of phytochemicals on GC and discuss the mechanism of action on gastric cancer, and also to show their bioavailability and therapeutic effect on gastric cancer. For the purpose of the review, we used keywords, including gastric cancer and phytochemicals, plant active ingredients, phytochemicals, chemical protection of plants, to retrieve relevant references from 2012 to 2022 in PubMed database. If there are too few references in some part, we will appropriately expand the time span of references.

## 2 Effects of phytochemicals on the occurrence and development of GC

Numerous epidemiological studies have demonstrated that the intake of phytochemicals is essential to the prevention and treatment of gastric cancer (Mao et al., 2020). GC is a multi-center, multi-step phenomenon, involving a variety of physiological and pathological processes. The effects of phytochemicals in the treatment and prevention of GC have been widely studied, and their mechanism of action has also been studied. We explored the influence of phytochemicals on the main physiological and pathological processes related to gastric cancer.

### 2.1 Inhibition of GC cell proliferation

**FIGURE 2:**
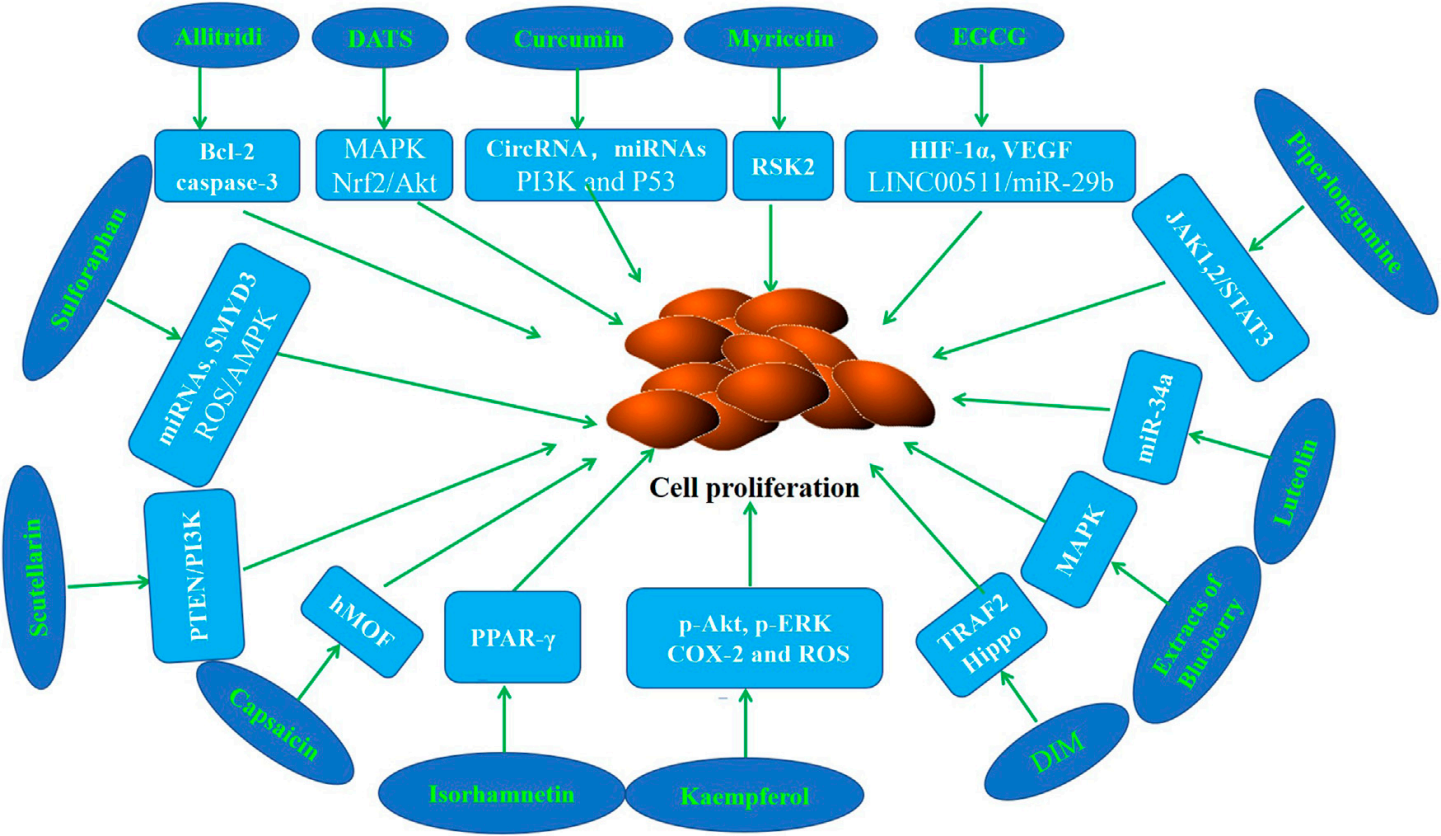
Molecular mechanism of anti-GC effect of representative phytochemicals by inhibiting cell proliferation.

Abnormal cell proliferation is a key step that may promote the occurrence and development of cancer (Kim et al., 2020a; Yang et al., 2020). Numerous studies have confirmed that various phytochemicals can inhibit the proliferation of GC cells and the growth of gastric tumors in mice (Table 1; Figure 2).

**TABLE 1:** Overview of the role of phytochemicals in the proliferation of gastric cancer.

Phytochemicals	Effects	Target	Subjects	Doses	References
Allitridi	Inhibited cell proliferation	Bcl-2, caspase-3	Gastric cancer cells	25 mg/L	13
Allitridi	Inhibited cell proliferation	p21	Gastric cancer cells	6 or 9 μg/mL	14
DATS	Inhibited cell proliferation	MAPK	Vivo and *in vitro* models	50, 100, 200 μM; 20, 30 and 40 mg/kg	15
DATS	Inhibited cell proliferation	Nrf2/Akt and p38/JNK	Vivo and *in vitro* models	50, 100, 200 μM; 20, 30 and 40 mg/kg	16
Curcumin	Inhibited cell proliferation	Circ0056618/miR-194-5p	Gastric cancer cells	20 μg/mL	17
Curcumin	Inhibited cell proliferation	miRNA-21	Gastric cancer tissues and cells	30 μmol/L	18
Curcumin	Inhibited cell proliferation	miR-34a	Gastric cancer cells	50 μM	19
Curcumin	Inhibited cell proliferation	PI3K and P53	Gastric cancer cells	20 µM	20
Curcumin	Inhibited cell proliferation	ATP-sensitive potassium channel	Gastric cancer cells	15, 30, 60 μM	21
Curcumin	Inhibited cell proliferation	ROS-mediated DNA polymerase γ depletion	Gastric cancer cells	10 μg/mL	22
Poncirin	Inhibited cell proliferation	—	Gastric cancer cells	5–25 μg/mL	23
Myricetin	Inhibited cell proliferation	RSK2	Gastric cancer cells	40 μmol/L	24
EGCG	Inhibited cell proliferation	HIF-1α and VEGF	Gastric cancer cells	20, 60, 100 μg/mL	25
EGCG	Retarded cell growth	LINC00511/miR-29b/KDM2A	Gastric cancer cells	100 μmol/L	26
Piperlongumine	Suppressed cell proliferation	JAK1,2/STAT3	Gastric cancer cells	10, 20, 40 µM	27
Kaempferol	Suppressed cell proliferation	p-Akt, p-ERK and COX-2	Vivo and *in vitro* models	60 or 120 µM	28
Kaempferol	Suppressed cell proliferation	Excessive ROS	Gastric cancer cells	25–100 μg/mL	29
DIM	Inhibited cell proliferation	TRAF2	Gastric cancer cells	80 µM	30
DIM	Inhibited cell proliferation	Hippo pathway	Vivo and *in vitro* tumor models	100 µM	31
Luteolin	Decreased viability of cells	miR-34a	Gastric cancer cells	5, 10 and 50 μM	32
Quercetin	Inhibited cell growth		Gastric cancer cells	40–200 μmol/L	33
Galangin	Inhibited cell growth		Gastric cancer cells	160 μmol/L	33
Isorhamnetin	Inhibited cell proliferation	PPAR-γ	Vivo and *in vitro* tumor models	25 µM	34
Ellagic acid	Inhibited cell proliferation	P53, BAX, APAF1, BCL2, iNOS, NF-κB, IL-8, TNF-α	Vivo and *in vitro* tumor models	15 and 30 μg/mL	4
Sulforaphan	Suppressed GC growth and cell proliferation	miR-29a-3p	Vivo and *in vitro* tumor models	12 μM	35
Sulforaphan	Inhibited cell proliferation	miR-9 and miR-326	Gastric cancer cells	250 μg/mL	36
Sulforaphan	Inhibited cell growth	ROS/AMPK	Gastric cancer cells	20 µM	37
Sulforaphan	Inhibited cell proliferation	SMYD3	Gastric cancer cells	2, 8, 32 µM	38
Leaf Extracts of Blueberry Plants	Inhibited cell proliferation	MAPK	Gastric cancer cells	0–3200 μg/mL	39
Capsaicin	Inhibited cell growth	hMOF	Gastric cancer cells	0–10 μg/mL	40
Scutellarin	Inhibited cell growth	PTEN/PI3K	Gastric cancer cells	10 µM	41

It has been shown by epidemiological evidence that Allitridi reduces the risk of developing malignancies (Sarvizadeh et al., 2021; Rauf et al., 2022). Several studies revealed that Allitridi and Diallyl trisulfide (DATS) inhibit cell proliferation in GC cell lines (Lan and Lu, 2004; Ha et al., 2005; Jiang et al., 2017a). Diallyl trisulfide suppressed tumor growth through the attenuation of Nrf2/Akt and activation of p38/JNK in xenograft mice (Jiang et al., 2017b). Curcumin has garnered attention because of its antiinflammatory, antioxidant, anticancer, and chemopreventive properties. It is reported that curcumin suppresses the proliferation of GC cells by regulating circRNA/miRNA/protein *in vivo* and *in vitro* experimental models (Liu et al., 2014; Wang et al., 2017; Fu et al., 2018; Liu et al., 2018; Hassanalilou et al., 2019; Sun et al., 2019). Poncirin is a flavanone glycoside that could inhibit the proliferation of SGC-7901 cells (Zhu et al., 2013). Myricetin is a flavonoid which could inhibit the abnormal proliferation of GC cells by binding with RSK2 (Feng et al., 2015). Epigallocatechin-3-gallate (EGCG), the most abundant and active polyphenol in green tea, has been shown to have anti-inflammatory, anti-oxidant, anti-cancer, and chemopreventive properties. Fu et al. (2019) revealed that EGCG down regulated HIF-1α and VEGF to inhibit the proliferation of GC cells. The data of Zhao et al. (2020) showed that EGCG retarded cell growth of GC in a dose-dependent manner. Piperlongumine, a major component derived from long peppers, has been reported to suppress the proliferation of GC cells (Song et al., 2016). It is reported that kaempferol inhibits the proliferation of GC cell lines and the growth of the tumor xenografts (Song et al., 2015; Liao et al., 2016). Recent studies have revealed that 3,3-diindolylmethane (DIM) has antiproliferation effects *in vivo and in vitro* GC models (Li et al., 2013; Ye et al., 2021a). Luteolin is a compound of Lonicera japonica Thunb, and has been reported to decrease the viability of cells in the occurrence and development of gastric cancer (Zhou et al., 2018). The study of Xu et al. (2017) reported that the growth inhibition of Galangin and quercetin on the GC cells . Lalitha *et al*. reported that isorhamnetin inhibits cell proliferation through the modulation of PPAR-γ activation in gastric cancer (Ramachandran et al., 2012). Data of Hamid et al. showed that Elagic acid inhibits the proliferation of GC cells and leads to the reduction of tumor volume in mice (Cheshomi et al., 2022). Sulforaphane is a natural compound of cruciferous vegetables. Sholeh et al. found that significant dose-dependent antiproliferative effects of sulforaphane were observed in GC cells (Choi, 2018; Dong et al., 2018; Kiani et al., 2018; Han et al., 2021). The study of Alejandra *et al.* demonstrated that the antiproliferative effect of leaf extracts of blueberry plants on GC cells (Ribera-Fonseca et al., 2020). The results of Wang et al. (2016a) showed that capsaicin could suppress cell growth, while changing histone acetylation in GC cells. Scutellarin was found to inhibit GC cell proliferation (Li et al., 2021a). Unfortunately, most of these studies focus on the anti-proliferation study of phytochemicals at the cell line level, and the dosage used is inconsistent, resulting in limited clinical value.

Uncontrolled proliferation of GC cells has been proved to play a critical role in the pathogenesis of gastric cancer. It is generally believed that some phytochemicals possess good effects on cancer prevention and growth. In recent years, there have been many studies involving the inhibition of cell proliferation by phytochemicals in the carcinogenesis and development of gastric cancer. These findings suggested that phytochemicals can be used as a potential means for the prevention and treatment of gastric cancer.

### 2.2 Inhibition of cell migration and invasion

The ability of cell migration and invasion plays an important role in the occurrence, development, treatment and prognosis of gastric cancer. Some GC patients have lymph node metastasis or even distant metastasis at the first diagnosis, which leads to failure of surgical treatment and affects the prognosis and survival rate of patients (Guo et al., 2021). The enhanced motility and invasiveness afforded by EMT are critical for metastatic initiation of gastric cancer (Li et al., 2019). There is increasing evidence that phytochemicals can inhibit the migration and invasion of GC cells *in vivo* and *in vitro* (Table 2).

**TABLE 2:** Overview of the role of phytochemicals in cell migration and invasion.

Phytochemicals	Effects	Target	Subjects	Doses	References
Curcumin	Suppressed cell migration and invasion	MAPK	Gastric tissue of mice	50 or 100 mg/kg	44
Curcumin	Suppressed cell migration and invasion	Gli1-β-catenin	Gastric cancer cells	30 µM	45
Curcumin	Suppressed cell migration and invasion	circ0056618/miR-194-5p	Gastric cancer cells	30 µM	46
Curcumin	Suppressed cell migration and invasion	miRNA-21	Gastric cancer cells	30 μmol/L	18
Curcumin	Inhibited cell metastasis	CXCR4	Gastric cancer cells	0.5 μmol/L	47
Isorhamnetin	Inhibited cell migration and invasion	PPAR-γ	Vivo and *in vitro* models	25 µM	34
Scutellarin	Inhibited cell migration and invasion	PTEN/PI3K	Gastric cancer cells	10 µM	41
EGCG	Inhibited cell migration and invasion	ERK5	Gastric tissue of mice	50 or 100 mg/kg	5
Hesperetin	Inhibited cell migration and invasion	DOT1L and histone H3K79	Gastric cancer cells	100 μM	48
Astragalin	Inhibited cell migration and invasion	PI3K/AKT	Gastric cancer cells	10, 20, 40 and 80 µM	49
Luteolin	Suppressed cell migration and invasion	Notch	Vivo and *in vitro* models	30 µM	50
β-carotene	Suppressed cell migration and invasion	Notch	Gastric tissue of mice	10 mg/kg	51
Quercetin	Suppressed cell migration and invasion	uPA/uPAR	Gastric cancer cells	10 µM	52
Ellagic acid	Inhibited cell migration and invasion	MMP-2 and MMP-9	Vivo and *in vitro* models	15 and 30 μg/mL	4
Ellagic Acid	Inhibited cell migration and invasion	MMP7 and MMP9	Gastric cancer cells	5 and 10 µM	53
Sulforaphane	Inhibited cell invasion	MMP9, ROS/MAPK	Gastric cancer cells	10, 30, 50 µM	54
Sulforaphane	Inhibited cell migration	Bax/Bcl2, MAPK	Gastric cancer cells	1.5 μg/mL	55
Sulforaphane	Inhibited cell migration	SMYD3	Gastric cancer cells	2, 8, 32 µM	38
Leaf extracts of blueberry plants	Inhibited cell proliferation	MAPK	Gastric cancer cells	0–3200 μg/mL	39

Curcumin, the major active compound of the plant Curcuma longa, has been shown to inhibit migration and invasion of GC cells (Liang et al., 2015; Liu et al., 2018; Zhang et al., 2020; Li et al., 2021b). The study of Gu et al. (2019) suggested that curcumin inhibits liver metastasis of GC through reducing circulating cancer cells. Lalitha et al. reported that isorhamnetin inhibits cell migration and invasion through the modulation of PPAR-γ activation in gastric cancer (Ramachandran et al., 2012). Scutellarin, a flavonoid plant compound derived from breviscapus, has been found to suppress GC cell migration and invasion (Li et al., 2021a). EGCG suppressed ERK5 activation to reverse tobacco smoke-triggered cell migration and invasion in mice gastric tissues (Lu et al., 2016). The author explored the intervention effect of EGCG in smoke induced GC *in vivo* and *in vitro*, which is still an interesting study. Hesperidin decreased the migration and invasion of GC cells by educing the abundance of DOT1L and methylation of histone H3K79 (Wang et al., 2021a). It is reported that Astragalin, a natural flavonoid compound, suppresses GC cells migration and invasion (Wang et al., 2021b). Luteolin significantly inhibited GC cells invasion and migration in a dose-dependent manner *via* the Notch pathway (Zang et al., 2017a). β-carotene, the carotenoid in fruits and vegetables, suppressed tobacco smoke-triggered cell migration and invasion in mice gastric tissues (Lu et al., 2018). Quercetin inhibited GC cells invasion and migration *via* the interruption of uPA/uPAR function (Li and Chen, 2018). Study of Hamid and Lim et al. (2019) found that Elagic acid inhibits the invasion and migration of GC cells *in vivo* and *in vitro* (Cheshomi et al., 2022). Sulforaphane is a phytochemical found in many cruciferous vegetables. Studies have showed that sulforaphane inhibits cell invasion and migration in human GC cells (Mondal et al., 2016; Dong et al., 2018; Li et al., 2022). The results of Alejandra et al. demonstrated that leaf extracts of blueberry plants suppress the migration of GC cells *in vitro* (Ribera-Fonseca et al., 2020).

More and more studies showed that phytochemistry can inhibit cell migration and invasion in the process of gastric carcinogenesis and development. These findings suggested that phytochemistry has a good application prospect in the occurrence, progression, prognosis and recurrence of gastric cancer.

### 2.3 Regulation of cell apoptosis and autophagy

**FIGURE 3:**
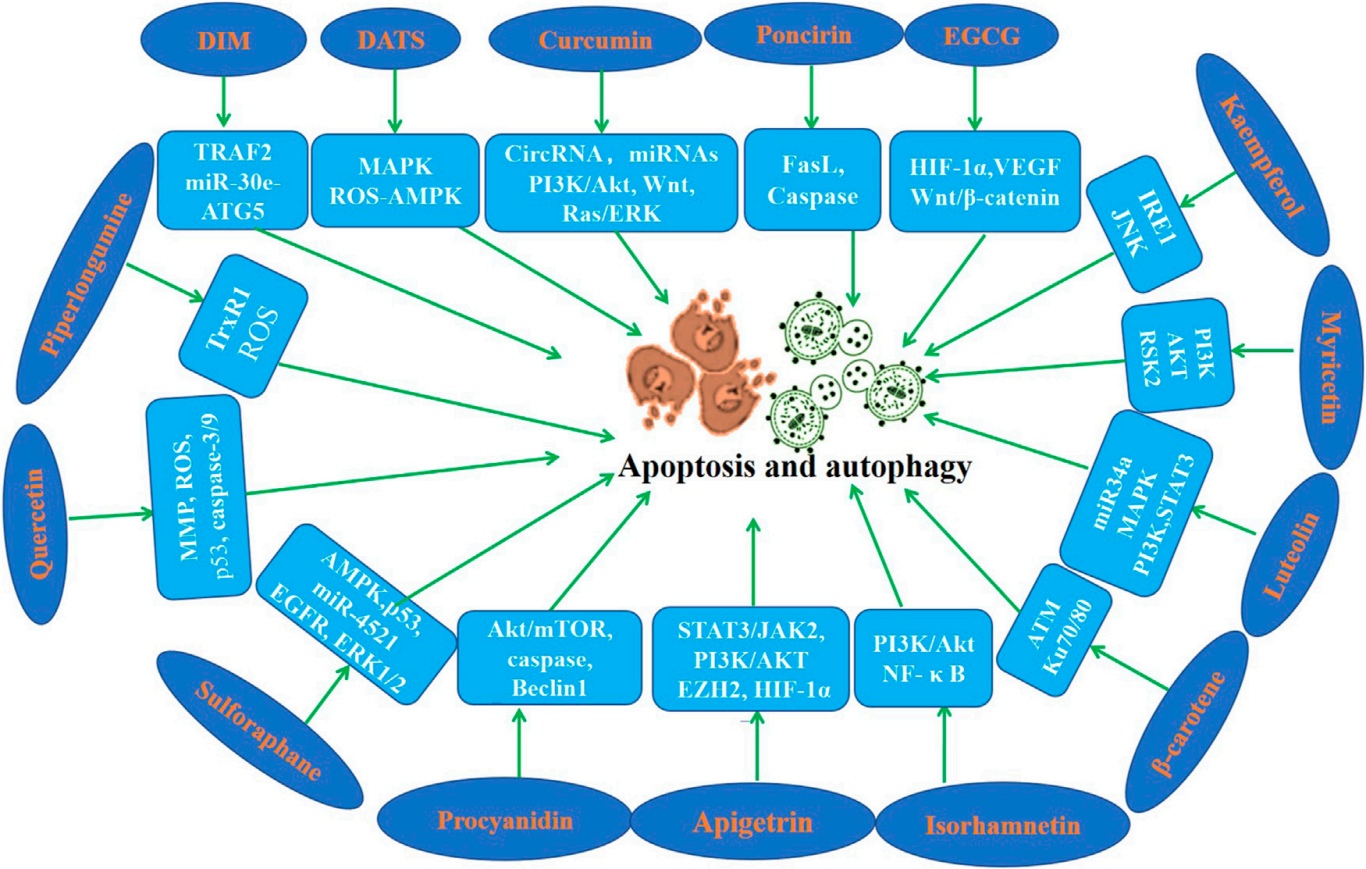
Molecular mechanism of anti-GC effect of representative phytochemicals by regulating apoptosis and autophagy.

Apoptosis is a highly regulated process of cell death. A series of studies using apoptosis have been proved to be effective in the prevention and treatment of many diseases including cancer (Pistritto et al., 2016; Xu et al., 2019; Berthenet et al., 2020). Cell autophagy is a highly conserved self-defense mechanism (Lu et al., 2022). Autophagy plays a key role in the occurrence, development and prognosis of GC (Cao et al., 2019; Wu et al., 2021; Lu et al., 2022). Induction of cell apoptosis and autophagy has been found maybe a pivotal mechanism of the inhibition of the initiation and the development of gastric cancer. In this section, we focus on the regulatory effects of phytochemicals on apoptosis and autophagy (Table 3; Figure 3).

**TABLE 3:** Overview of the role of phytochemicals in cell apoptosis and autophagy.

Phytochemicals	Effects	Target	Subjects	Doses	References
DATS	Promoted cell apoptosis	MAPK	Vivo and *in vitro* models	50, 100, 200 μM; 20, 30, 40 mg/kg	15
DATS	Promoted cell apoptosis	ROS-AMPK	Gastric cancer cells	50 μM	62
Curcumin	Promoted cell apoptosis	Circ0056618/miR-194-5p	Gastric cancer cells	30 μM	46
Curcumin	Promoted cell apoptosis	PI3K/Akt/mTOR	Gastric cancer cells	15, 20 μM	63
Curcumin	Promoted cell apoptosis	MiR-21/PTEN/Akt	Gastric cancer cells	20 μM	64
Curcumin	Promoted cell apoptosis	PI3K and P53	Gastric cancer cells	20 μM	20
Curcumin	Promoted cell apoptosis	Wnt/β-catenin	Gastric cancer cells	0–32 μM	65
Curcumin	Promoted cell apoptosis	Bcl-2 and Bax	Gastric cancer cells	5, 10, 20 μM	66
Curcumin	Promoted cell apoptosis	Ras/ERK	Gastric cancer cells	20 μM	67
Apigetrin	Promoted cell apoptosis	STAT3/JAK2	Gastric cancer cells	50 μM	68
Apigetrin	Promoted cell apoptosis	Mitochondrial pathway	Gastric cancer cells	10 μg/mL	69
Apigenin	Promoted apoptotic cell death	EZH2, HIF-1α	Gastric cancer cells	50 μM	70
Apigenin	Promoted apoptotic cell death	PI3K/AKT/mTOR	Gastric cancer cells	25, 50, 100 μM	71
Poncirin	Promoted cell apoptosis	FasL, Caspase-8, Caspase-3	Gastric cancer cells	50, 150 μM	72
Myricetin	Promoted cell apoptosis	PI3K/AKT/mTOR	Gastric cancer cells	15 μM	73
Myricetin	Promoted cell apoptosis	RSK2	Gastric cancer cells	20 or 40 μmol/L	24
EGCG	Increased cell apoptosis	HIF-1α and VEGF	Gastric cancer cells	100 μg/mL	25
EGCG	Increased cell apoptosis	wnt/β-catenin	Gastric cancer cells	30 μM	74
Hesperetin	Increased cell apoptosis	Intracellular ROS	Gastric cancer cells	200 μM	75
α-mangostin	Increased cell apoptosis	Stat3	Gastric cancer cells	7 μg/mL	76
Piperlongumine	Induced cell apoptosis	ROS	Vivo and *in vitro* models	7.5 μM	77
Piperlongumine	Induced cell apoptosis	TrxR1	Vivo and *in vitro* tu models	15 μM	78
p-Coumaric acid	Induced cell apoptosis	miR-125a-5p, miR-30a-5p, miR-7-5p	Gastric cancer cells	1.5 mM	79
Astragalin	Induced cell apoptosis	PI3K/AKT	Vivo and *in vitro* models	10, 20 or 40 μM	49
DIM	Induced cell apoptosis	TRAF2	Gastric cancer cells	20, 40, 60 or 80 μM	30
Luteolin	Induced cell apoptosis	miR-34a	Gastric cancer cells	40 μM	80
Luteolin	Induced cell apoptosis	STAT3	Gastric cancer cells	10 μM	81
Luteolin	Induced cell apoptosis	MAPK and PI3K	Gastric cancer cells	20, 40 and 60 µM	82
Zerumbone	Induced cell apoptosis	Cyp A	Gastric cancer cells	12.27 μM	83
β-carotene	Promoted cell apoptosis	ATM	Gastric cancer cells	100 μmol/L	84
β-carotene	Promoted cell apoptosis	Ku70 and Ku80	Gastric cancer cells	100 μM	85
Procyanidin	Induced cell apoptosis	Akt/mTOR	Gastric cancer cells	20, 50 and 100 μM	86
Procyanidin	Induced cell apoptosis	Beclin1 and BCL-2	Gastric cancer cells	40.7 μg/mL	87
Quercetin	Induced cell apoptosis	p53, caspase-3, -9, and Parp	Xenograft Models	30 mg/kg/day	88
Quercetin	Induced cell apoptosis	ROS	Gastric cancer cells	160 μM	89
Quercetin	Induced cell apoptosis	MMP, caspase-3, -9	Gastric cancer cells	40–200 μmol/L	33
Isorhamnetin	Promoted cell apoptosis	PI3K	Gastric cancer cells	28 μmol/L	90
Isorhamnetin	Promoted cell apoptosis	PI3K/Akt and NF- κ B	Gastric cancer cells	100 μmol/L	91
Sulforaphane	Induced cell apoptosis	AMPK	Gastric cancer cells	20 μM	37
Sulforaphane	Induced cell apoptosis	miR-4521/PIK3R3	Gastric cancer cells	1.5 μg/mL	55
Sulforaphane	Induced cell apoptosis	p53	Gastric cancer cells	5 and 10 μM	92
Lycopene	Induced cell apoptosis	β-catenin	Gastric cancer cells	.5, 1, and 2 µM	93
Procyanidin	Augmented cell apoptosis	caspase-3 and -9	Gastric cancer cells	200 μg/mL	94
Capsaicin	Promoted cell apoptosis	p53	Gastric cancer cells	200 mM	95
Eugenol	Promoted cell apoptosis	—	Gastric cancer cells	.7 mM	95
Apigenin	Promoted autophagic cell death	PI3K/AKT/mTOR	Gastric cancer cells	25, 50 and 100 μM	71
DIM	Inhibited cell autophagy	miR-30e-ATG5	Vivo and *in vitro* models	60 μM	96
Perilaldehyde	Induce cell autophagy	AMPK	Gastric cancer cells	1 mM	97
Sulforaphane	Suppressed cell autophagy	EGFR, p-ERK1/2	Gastric cancer cells	2, 3.5 and 5.5 μg/mL	55
Sulforaphane	Suppressed cell autophagy	p53	Gastric cancer cells	5 and 10 μM	92
Sulforaphane	suppressed cell autophagy	miR-4521/PIK3R3	Gastric cancer cells	10, 20 and 50 μM	98
Procyanidin	Induced cell autophagy	Akt/mTOR	Gastric cancer cells	20, 50 and 100 μM	86
Procyanidin	Induced cell autophagy	Beclin1 and BCL-2	Gastric cancer cells	40.7 μg/mL	87
Isorhamnetin	Promoted cell autophagy	PI3K	Gastric cancer cells	10 μmol/L	90
Kaempferol	Induced autophagic cell death	IRE1/JNK/CHOP	Gastric cancer cells	50 μM	99

DATS has shown its excellent anti GC effect in various studies. DATS promoted cell apoptosis of GC cells *in vivo* and *in vitro* (Jiang et al., 2017a; Choi, 2017). Numerous studies have shown that curcumin promotes cell apoptosis of GC cells by regulating circRNA/miRNA/protein *in vivo* and *in vitro* (Xue et al., 2014; Cao et al., 2015; Li et al., 2017; Zheng et al., 2017; Fu et al., 2018; Qiang et al., 2019; Li et al., 2021b). However, the bioavailability of curcumin has always been an urgent problem to be solved. We need to find better drug delivery methods, such as nano vesicles or exosomes, which may improve the bioavailability of curcumin. Apigenin enhanced cell apoptosis of GC cells in a time and dose-dependent manner (Chen et al., 2014; Sun et al., 2018). Findings of Seong and Chen et al. indicated that Apigetrin activates apoptotic cell death *via* HIF-1α, Ezh2 and PI3K/AKT/mTOR in GC cells (Kim et al., 2020b; Kim and Lee, 2021). Poncirin exists in many citrus fruits, and it has been found that it can promote AGS cell apoptosis and play an anti-cancer role (Saralamma et al., 2015). Myricetin is a natural flavonoid found in berries, green tea and nuts, which induces apoptosis of GC cells and exerts anti-GC effects (Feng et al., 2015; Han et al., 2022). Studies demonstrated that EGCG induced GC cells apoptosis in a dose-dependent manner (Yang et al., 2016; Fu et al., 2019). Zhang *et al.* suggested that hesperidin induces GC cells apoptosis *via* by increasing the ROS (Zhang et al., 2015). α-Mangosterin, a major xanthone found in the pericarp of mangosteen, can significantly promote apoptosis of GC cells (Shan et al., 2014). Piperlongumine is a natural alkaloid, which induced GC cell apoptosis *in vitro* and *in vivo* (Duan et al., 2016; Zou et al., 2016). P-coumaric acid is a phenolic compound abundant in edible plants, which was found to induce apoptosis of GC cells (Jang et al., 2020). It is reported that Astragalin induces apoptosis of GC cells and then exerts its anticancer activity (Wang et al., 2021b). Study have revealed that DIM induced apoptosis of GC cells (Ye et al., 2021a). Luteolin is a natural flavonoid that exists in vegetables, fruits and medicinal herbs, which promotes GC cells apoptosis (Wu et al., 2015; Lu et al., 2017; Song et al., 2017). Zerumbone could induce apoptosis of GC cells through down-regulating CypA (Wang et al., 2016b). Studies found that β-carotene induces apoptosis in AGS cells (Jang et al., 2009; Park et al., 2015). Proanthocyanidins are flavonoids widely found in the skin and seeds of various plants, which have been found to induce apoptosis of GC cells (Nie et al., 2016; Li et al., 2021c). Quercetin is a natural component of natural plants, which induced apoptosis of GC cells *in vivo* and *in vitro* (Lee et al., 2016; Xu et al., 2017; Shang et al., 2018). Isorhamnetin induced GC cells apoptosis through PI3K, Akt and NF-κB pathways (Duan et al., 2020; Li et al., 2021d). Sulforaphane significantly enhanced GC cells apoptosis in a dose-dependent manner (Mondal et al., 2016; Choi, 2018; Wang et al., 2021c). Lycopene induced GC cells apoptosis by inhibiting nuclear translocation of β-catenin (Kim et al., 2019a). Anthocyanins isolated from Vitis coignetiae, augmented GC cells apoptosis by activating caspase-3 and caspase-9 (Park et al., 2021). Capsaicin and eugenol induced GC cells apoptosis in the presence or absence of functional p53 (Sarkar et al., 2015). Choi *et al*. reported that sulforaphane induced GC cells apoptosis by mediating activation of AMPK (Choi, 2018).

According to Seong and colleagues, Apigetrin increased autophagic cell death *via* HIF-1α, Ezh2 and PI3K/AKT/mTOR in GC cells (Kim et al., 2020b). Ye et al. (2016) reported a novel regulation of GC cells autophagy by DIM *in vivo* and *in vitro* models. Perilaldehyde induced autophagy in GC cells and inhibited the growth of gastric cancer (Zhang et al., 2018). Isorhamnetin induced GC cells autophagy *via* the PI3K pathway (Li et al., 2021d). Sulforaphane also suppressed cell autophagy during the progression of gastric cancer (Mondal et al., 2016; Wang et al., 2021c; Peng and Gu, 2021). Procyanidin exerted anti-cancer activity in GC by regulating autophagy (Nie et al., 2016; Li et al., 2021c). The findings of Tae et al. indicated that kaempferol activates the IRE1/JNK/CHOP signaling to induce autophagic cell death in GC cells (Kim et al., 2018).

Taken together, these findings above illustrated that phytochemistry might be used as a promising candidate against the initiation and progression of GC by mediating cell apoptosis and autophagy.

### 2.4 Enhancement on chemosensitivity in GC

Although great progress has been made in the study of the mechanism of occurrence and development of GC in recent years, surgery with or without chemotherapy is still the appropriate treatment strategy for gastric cancer. However, resistance has become a major problem in the treatment of gastric cancer. In this chapter, we mainly discuss the role of phytochemistry in enhancing the sensitivity of cells to chemotherapy drugs (Table 4).

**TABLE 4:** Overview of the effect of phytochemicals on chemosensitivity.

Phytochemicals	Effects	Target	Chemotherapy drug	Doses	References
DATS	Enhanced chemosensitivity	Nrf2/Akt and p38/JNK	Cisplatin	50–200 μmol/L	16
DATS	Enhanced chemosensitivity	NF-κB	Docetaxel	40 μM	100
Curcumin	Enhanced chemosensitivity	JAK/STAT3	5-fluorouracil	20 μM	101
Curcumin	Enhanced chemosensitivity	COX-2 and NF- κB	5-fluorouracil	25 μmol/L	102
Curcumin	Enhanced chemosensitivity	Bcl/Bax-caspase3, 8,9	5-Fluorouracil and Oxaliplatin	10 μM	103
Curcumin	Enhanced chemosensitivity	NF- κB	5-fluorouracil	20 μM	104
EGCG	Enhanced chemosensitivity	p19Arf-p53-p21Cip1	Cisplatin	25 μg/mL	105
Protocatechuic Acid	Enhanced chemosensitivity	p53	5-fluorouracil	500 μM	106
α-mangostin	Enhanced chemosensitivity	EBI3/STAT3	Cisplatin	15 μM	107
Piperlongumine	Enhanced chemosensitivity	ROS	Oxaliplatin	4 μM	108
DIM	Enhanced chemosensitivity	Akt/FOXM1	Paclitaxel	50 μM	109
Luteolin	Enhanced chemosensitivity	Cyt c/caspase	Oxaliplatin	40 μM	110
Quercetin	Enhanced chemosensitivity	VEGF	Irinotecan and its metabolite, SN-38	12.5 μM	111
Quercetin	Enhanced chemosensitivity	NF- κB	5-fluorouracil and adriamycin	25 μM	112
Isorhamnetin	Enhanced chemosensitivity	NF-κB	Capecitabine	50 μM	113
Sulforaphane	Enhanced chemosensitivity	HER-2, AKT, ERK	Lapatinib	5 μM	114
Sulforaphane	Enhanced chemosensitivity	miR-124/IL-6R/STAT3	Cisplatin	10 μM	115
[6]-Gingerol	Enhanced chemosensitivity	PI3K/AKT	Cisplatin	300 μM	116
Anthocyanins	Enhanced chemosensitivity	PI3K/AKT	Cisplatin	200 μM	117
Liquiritin	Enhanced chemosensitivity	CDK4, p53 and p21	Cisplatin	80 μM	118
Astragalus polysaccharide	Enhanced chemosensitivity	AKT	Apatinib	200 μg/mL	119
Tanshinone IIA	Enhanced chemosensitivity	miR-125a-5p, miR-30a-5p, miR-7-5p	Gastric cancer cells	5 μM	120

Studies provided evidences that DATS enhances the sensitivity of GC cells to cisplatin and docetaxel, meanwhile DATS exerts excellent anticancer effects (Pan et al., 2016; Jiang et al., 2017b). Curcumin has shown excellent anticancer effects in a variety of tumors. Studies have found that curcumin enhances the sensitivity of GC cells to first-line chemotherapy drugs such as 5-fluorouracil and oxaliplatin *in vitro* and *in vivo* (Kang et al., 2016; Zhou et al., 2016; Yang et al., 2017; Ham et al., 2022). EGCG enhanced the effect of cisplatin on inhibiting GC cells proliferation and inducing cell apoptosis (Xue et al., 2021). Zhang et al. (2019) indicated that protocatechuic acid reduces the dosage of 5-fluorouracil and enhances the chemosensitivity of GC cells to 5-fluorouracil (Motamedi et al., 2020). It is reported that α-mangostin increases the chemosensitivity of GC cells to cisplatin by inactivating the EBI3/STAT3 pathway (Li and Zeng, 2021). These data of Zhang et al. (2019) demonstrated that piperlongumine potentiates the effect of chemotherapy of oxaliplatin in GC cells. The findings of Jin and Park et al. suggested that DIM improves the efficacy of paclitaxel through the Akt/FOXM1 in gastric cancer (Jin et al., 2015). It is elucidated that luteolin potentiated the sensitivity of GC cells to Oxaliplatin through Cytc/caspase (Ren et al., 2020). Studies investigated that quercetin enhances the therapeutic effect of irinotecan/SN-38, 5-fluorouracil and Adriamycin in gastric cancer (Hyun et al., 2018; Lei et al., 2018). Kanjoormana et al. demonstrated that isorhamnetin enhances the anti-GC effects of capecitabine through the NF-κB pathway (Manu et al., 2015). It is reported that sulforaphane might be a promising therapeutic treatment for lapatinib-resistant and cisplatin-resistant gastric cancer (Wang et al., 2016c; Yi et al., 2021). Cisplatin based chemotherapy is a widely used chemotherapy regimen for gastric cancer, [6]-gingerol enhances the sensitivity of GC cells to cisplatin (Luo et al., 2019). Results suggested that anthocyanins enhance anti-GC effects of Cisplatin *via* inhibiting Akt activity (Lu et al., 2015). Liquiritin circumvented the resistance of cisplatin in cisplatin-resistant GC cells (Wei et al., 2017). Astragalus polysaccharide was reported to enhances the antitumor effects of Apatinib in GC cells (Wu et al., 2018). It is found that tanshinone IIA enhanced the anticancer effect of doxorubicin on drug-resistant GC cells (Xu et al., 2018). Some phytochemicals may exhibit excellent anti-cancer activity in cell and animal research, but their clinical application will be limited because the plants from which these phytochemicals come are uncommon or our body cannot take them regularly.

### 2.5 Suppression of GC stem cells properties

GC stem cells are a kind of cells with self-renewing and multi-directional differentiation ability. GC stem cells play an critical role in the occurrence, development, heterogeneity, drug resistance, metastasis and recurrence of GC (121, 122). In this chapter, we aim to explore whether phytochemicals can modulate the stemness of GC stem cells to induce a tumorigenic effect.

Ge et al. (2019) found that sulforaphane suppresses the stemness of GC stem cells by inhibiting the Hedgehog pathway. It is reported that Apatinib suppresses GC stem cells properties *via* inhibiting the Hedgehog pathway (Cao et al., 2021). Low levels of DIM promoted GC progression by activating the Wnt4 pathway to enhance GC cell stemness (Zhu et al., 2016). Sulforaphane regulated GC stem cell properties through the miR-124/IL-6R/STAT3 axis (Wang et al., 2016c). The results of Shen et al. (2016) demonstrated that quercetin inhibits the growth of GC stem cells by inhibiting PI3K/Akt signaling. Constantly exploring phytochemistry that can inhibit stem cell stemness may be a new strategy for prevention and treatment of GC patients with drug resistance, radiotherapy insensitivity and poor prognosis.

### 2.6 Inhibition of angiogenesis and lymphangiogenesis

Accumulating evidence showed that angiogenesis and lymphangiogenesis play an important role in the occurrence, progression and metastasis of gastric cancer (Da et al., 2015; Zang et al., 2017b; Huang et al., 2017; Da et al., 2019). Studies have found that phytochemicals can prevent and treat GC by inhibiting angiogenesis and lymphatic lineation (Da et al., 2015; Zang et al., 2017b; Huang et al., 2017; Da et al., 2019). Herein, we summarized phytochemicals that inhibit angiogenesis, lymphangiogenesis and analyzed the molecular mechanisms.

It is reported that curcumin inhibits gastric cancer-derived MSC mediate angiogenesis through regulating the NF-κB/VEGF pathway (Huang et al., 2017). Luteolin suppressed angiogenesis by inhibiting the Notch1/VEGF pathway in gastric cancer (Zang et al., 2017b). Tsuboi et al. (2014) found that zerumbone suppresses tumor angiogenesis in gastric cancer. Nitinodine chloride, a natural phytochemical alkaloid, could significantly inhibit angiogenesis of GC *in vivo* and *in vitro* (Chen et al., 2012). Curcumin suppressed the lymphangiogenesis of GC cells *in vivo* and *in vitro* (Da et al., 2015; Da et al., 2019).

### 2.7 Modulation of microenvironment and microbiota

In recent years, the relationship between the gut microenvironment and GC has attracted more and more attention (Mao et al., 2020). It was reported that phytochemicals could manage cancers through the modulation of the microenvironment (Mao et al., 2020; Xu et al., 2020). Kim *et al.* found that β-carotene and lutein inhibit the inflammatory environment around GC cells and oxidative stress, thus preventing the progression of gastric cancer (Kim et al., 2011). Atnip et al. (2020) indicated that anthocyanins suppress the inflammatory environment around GC cells. Gut microbiota also plays an important role in the occurrence, development and prognosis of gastric cancer (Nagano et al., 2019; Qi et al., 2019). Lofgren et al. (2011) reported in 2011 that microbiota may be related to gastric cancer, because mice without specific pathogens are more prone to atrophic gastritis and GC than mice without bacteria. However, there are few reports on the anti-GC effect of phytochemicals through regulating gut microbiota, which may require further elucidation and research.

### 2.8 Phytochemicals in screening phytochemistry targeting Helicobacter pylori

Accumulating research has proved that Helicobacter pylori infection causes some diseases in stomach and gastric cancer are closely related with it (Kuo et al., 2014; Santos et al., 2015; Ray et al., 2021). Various phytochemicals have shown anti Helicobacter pylori infection efficacy and can be used to prevent the occurrence and development of gastric cancer (Sekiguchi et al., 2008; Haghi et al., 2017).

Santos et al. (2015) and Ray et al. (2021) reported curcumin has a significant intervention effect on the occurrence of GC induced by Helicobacter pylori infection (Haghi et al., 2017). Apigenin has a remarkable ability to inhibit *Helicobacter* pylori-induced atrophic gastritis and GC progression Apigenin could significantly inhibit the progression of atrophic gastritis and GC induced by Helicobacter pylori (Kuo et al., 2014). The research results of Iwona et al. showed that luteolin can be used for the treatment and prevention of GC infected by Helicobacter pylori (Radziejewska et al., 2021). Studies found that consumption of β-carotene-rich foods may be beneficial to prevent gastric disease induced by helicobacter pylori infection (Kang and Kim, 2017). Similarly, many studies have found that β-carotene has a good application prospect in preventing GC induced by Helicobacter pylori infection (Park et al., 2019a; Kim et al., 2019b; Bae et al., 2021). Quercetin has a protective effect on gastric diseases related to Helicobacter pylori infection (Haghi et al., 2017; Zhang et al., 2017). Lycopene and DATS also have the ability to resist Helicobacter pylori infection (Haghi et al., 2017; Park et al., 2019b).

### 2.9 Other possible mechanisms

In addition to the above-mentioned modes of action, some phytochemistry also plays a preventive or therapeutic role in the occurrence and development of GC through other modes or mechanisms. DTAS exerted an anticancer effect in GC by regulating the antioxidant enzyme sulfiredoxin (Wang et al., 2019). DATS interfered with the occurrence and development of GC by regulating the activities of quinone oxidoreductase1, FRalpha and calcyclin genes (Li et al., 2002; Kim et al., 2014). Curcumin suppressed GC by inducing DNA demethylation and inhibiting gastrin-mediated acid secretion (Zhou et al., 2017; Tong et al., 2020). Scutellarin suppressed GC by altering lactate dehydrogenase profile, DNA density, mucus content and acidity (Sun and Meng, 2022). Kaempferol, p-Coumaric acid, Astragalin and Tiliroside influence abnormal glycosylation of GC cells, so as to exert the anticancer effect (Radziejewska et al., 2022). DIM suppressed GC *via* mediated ferroptosis, store-operated calcium entry, gastric cancer-derived mesenchymal stem cells, endogenous hydrogen sulfide biosynthesis (Ye et al., 2020; Ye et al., 2021b; Shi et al., 2021; Ye et al., 2022). It is reported that phytochemicals showed anticancer properties against GC associated with tumor viral infections (Liskova et al., 2021; Sudomova et al., 2021).

## 3 Summary and the challenges

Phytochemicals, are bioactive compounds that are found in plants such as vegetables, fruits, Chinese herbal medicines, etc. They have elucidated the anticancer activity against GC by adjusting several mechanisms such as inhibitory actions on cell proliferation, migration and invasion, regulating apoptosis and autophagy, enhancing chemosensitivity and blocking infection of Helicobacter pylori. Among them, we found that some phytochemicals have excellent anti GC activity, which can play an intervention effect in multiple processes of gastric cancer, such as proliferation, apoptosis, autophagy, invasion, cancer stem cells properties regulation, helicobacter pylori infection, etc. These excellent phytochemicals include curcumin, sulforaphane, EGCG, DATS, DIM, β-carotene, quercetin, isorhamnetin, luteolin, which are worthy of our in-depth research and development to provide strategies for early prevention and treatment of gastric cancer.

There is not much of phytochemistry really used in clinic and most of phytochemicals that are used in clinic are in an auxiliary role. How to better enhance the function of phytochemicals in GC prevention and treatment is particularly prominent. On one hand, we should devote ourselves to developing effective and safe natural phytochemicals to against gastric cancer. On the other hand, we need to find a more efficient and safer delivery system for phytochemistry *in vivo*.

In future work, we might deliver phytochemicals through an exosome pathway to improve the bioavailability and targeting of phytochemistry. Or, we might extract phytochemical exosome to effect on GC cells to observe whether they can enhance the anticancer effect and bioavailability. To explore whether phytochemicals can interfere with the development of GC by changing the active components carried by exosomes of GC cells.

Maybe we should pay attention to several aspects in future research. Deliver phytochemicals through an exosome pathway to enhance the bioavailability and targeting of phytochemistry. Extract the exosomes of phytochemicals act on GC cells and observe whether they can enhance the anticancer effect and bioavailability. To explore whether phytochemicals can interfere with the development of GC by changing the active components carried by exosomes of GC cells.
