# Corrigendum: Anticancer applications of phytochemicals in gastric cancer: effects and molecular mechanism

**DOI:** 10.3389/fphar.2024.1405513

**Published:** 2024-08-19

**Authors:** Zhaofeng Liang, Yumeng Xu, Yue Zhang, Xinyi Zhang, Jiajia Song, Hui Qian, Jianhua Jin

**Affiliations:** ^1^ Wujin Institute of Molecular Diagnostics and Precision Cancer Medicine of Jiangsu University, Wujin Hospital Affiliated with Jiangsu University, Chang Zhou, China; ^2^ Department of Laboratory Medicine, School of Medicine, Jiangsu University, Zhenjiang, China

**Keywords:** gastric cancer, phytochemicals, prevention, Treatment, mechanisms

In the published article, there was an error in the **Author list**, and author Jianhua Jin appeared in the wrong order in the author list and in the corresponding authors list.

The corrected author list appears below.

Zhaofeng Liang^1,2*†^, Yumeng Xu^2†^, Yue Zhang^2^, Xinyi Zhang^2^, Jiajia Song^2^, Hui Qian^1,2*^ and Jianhua Jin^1*^.

The order of email addresses for corresponding authors is as follows:

Jianhua Jin, jianhuajin88@sina.com, Zhaofeng Liang, liangzhaofeng@ujs.edu.cn, Hui Qian, lstmmmlt@163.com.mailto:lstmmmlt@163.com

The authors apologize for this error and state that this does not change the scientific conclusions of the article in any way. The original article has been updated.

